# Does ^18^F-sodium fluoride PET/CT have a role in the assessment of response to ^223^Ra therapy in men with prostatic bone metastases?

**DOI:** 10.1007/s00259-026-08009-8

**Published:** 2026-06-20

**Authors:** Glen M. Blake, Ricardo Donners, Amelia E.B. Moore, Holly Tovey, Aude Espinasse, Matthew D. Blackledge, Sue Chua, Yong Du, Emma Hall, Dow-Mu Koh, Emilia Nuzzaci, Nina Tunariu, Christopher C. Parker, Gary J.R. Cook

**Affiliations:** 1 Department of Biomedical Engineering, School of Biomedical Engineering & Imaging Sciences, King’s College London, St Thomas’ Hospital, London, UK; 2 Department of Radiology, University Hospital Basel, Petersgraben 4, Basel, 4031 Switzerland; 3 Department of Cancer Imaging, School of Biomedical Engineering & Imaging Sciences, King’s College London, St Thomas’ Hospital, London, UK; 4 The Institute of Cancer Research, 15 Cotswold Road, Sutton, SM2 5NG UK; 5 Royal Marsden Hospital, Downs Road, Sutton, SM2 5PT UK

**Keywords:** Prostate cancer, Bone metastases, Radium-223 therapy, Diffusion-weighted MRI, ^18^F]NaF PET/CT imaging, Maximum standardised uptake values, Bone metabolic flux (Ki)

## Abstract

**Background:**

The REASURE study investigated imaging biomarkers in men receiving ^223^Ra treatment for prostate cancer bone metastases. We used REASURE data to compare and contrast the roles of [^18^F]NaF PET/CT measurements of maximum standardised uptake value (SUVmax) and bone metabolic flux (Ki) as markers of response.

**Methods:**

Thirty-four men with prostatic bone metastases received up to six cycles of ^223^Ra (55 or 88 kBq/kg) at four-weekly intervals. Whole-body diffusion-weighted MRI and [^18^F]NaF PET/CT images were acquired at baseline and 4, 12, and 24 weeks later. Values of the apparent diffusion coefficient (ADC, a measure of tumour response), SUVmax, and a novel surrogate measurement of Ki using a PET/CT SUV measurement in the left ventricle were monitored in up to 5 bone metastases in each participant. Multilinear regression analysis (MLR) was performed to determine if changes in ADC were predicted by changes in SUVmax or Ki. Radium dose and baseline SUVmax were additional independent variables.

**Results:**

ADC values increased throughout the study, while SUVmax and Ki decreased. MLR analysis showed that baseline SUVmax and ^223^Ra dose predicted ADC response (*P* < 0.001 and *P* < 0.01, respectively). Changes in SUVmax and Ki at 4, 12, and 24 weeks failed to predict the changes in ADC, showing decoupling between MRI and PET/CT measurements as markers of response. Changes in Ki were a highly significant predictor of changes in SUVmax (*P* < 0.001), reflecting the strong correlation between these two measurements.

**Conclusions:**

Baseline SUVmax was the best predictor of ADC response, followed by ^223^Ra dose. Changes in SUVmax and Ki failed to predict changes in ADC, suggesting that the changes in the PET/CT variables reflected the effect of ^223^Ra uptake on osteoblasts rather than a tumoricidal effect. A decrease in SUVmax seen on post-therapy [^18^F]NaF PET/CT scans should not be interpreted as evidence of tumour regression.

**Trial Registration:**

ISRCTN ISRCTN17805587. Registered 21/01/15.

**Supplementary Information:**

The online version contains supplementary material available at https://doi.org/10.1007/s00259-026-08009-8.

## Introduction

Radium-223 (half-life 11.4 days) is a therapeutic radionuclide that selectively binds to sites of increased bone formation and emits alpha particles with ranges up to 100 μm [1, 2]. The ALSYMPCA trial, a phase 3, randomised, double-blind, multinational study, compared the efficacy and safety of radium-223 administered intravenously as radium dichloride ([^223^Ra]RaCl_2_) versus placebo in 921 men with castration-resistant prostate cancer and bone metastases without spread to other organs [3]. This study and others conducted since have demonstrated that [^223^Ra]RaCl_2_, administered as a course of six injections at a dose of 50 kBq/kg given at four-week intervals, has a palliative effect on prostatic bone metastases with improved survival and fewer adverse events compared with placebo [4–10]. A large prospective study, the REASSURE study, has gathered real-world observational data on the long-term outcomes of radium-223 therapy in a large cohort of patients with bone-dominant metastatic castration-resistant prostate cancer [11].

The REASURE study (not to be confused with the REASSURE study) was a multicentre study that aimed to identify potential imaging response biomarkers following [^223^Ra]RaCl_2_ therapy in men with prostatic bone metastases [12, 13]. Thirty-nine men were enrolled from three oncology centres and randomised to receive up to six cycles of either 55 or 88 kBq/kg ^223^Ra given at four-week intervals. Whole-body diffusion-weighted MRI and [^18^F]-sodium fluoride ([^18^F]NaF) PET/CT images were acquired at baseline and 4, 12, and 24 weeks later. The MRI scans were used to measure the apparent diffusion coefficient (ADC) at sites of metastatic bone disease in each participant. MRI ADC measurements quantify the mobility of water molecules, correlate inversely with the cellularity of tumour tissue [12, 13], and are widely used as a reference standard for quantifying tumour viability and response to treatment [14–17]. The [^18^F]NaF PET/CT scans were used to measure the maximum standardised uptake values (SUVmax) [18] at the same sites as the ADC measurements. SUVmax measurements reflect the local osteoblastic activity in bone and an increase in the number of lesions or in their SUVmax values is usually taken as evidence of disease progression. However, a temporary increase in the number of lesions or their activity can also be caused by the bone scan flare phenomenon and may be indicative of a positive healing response [9, 19–22]. Initial results of the REASURE study were reported previously [12, 13].

Although SUVmax measurements are frequently used to quantify PET/CT studies in oncology [9, 18], in studies of metabolic bone diseases an alternative measurement of [^18^F]NaF tracer uptake, bone metabolic flux (Ki, also referred to as bone plasma clearance), is often reported [23–25]. Ki is measured in units of mL min^− 1^ mL^− 1^ and normalises the regional uptake of [^18^F]NaF in the bone volume of interest to the quantity of tracer delivered by arterial blood since the time of tracer injection [25]. Ki is thought to give a truer measure than SUVmax of the regional metabolic activity in bone [26]. Furthermore, a study in breast cancer patients with metastatic bone disease reported that Ki measurements more reliably identified those women with progressive disease than did SUVmax [27].

Here we report the results of a retrospective analysis of the REASURE study [^18^F]NaF PET/CT scans to measure the changes in SUVmax and Ki and compare their response to ^223^Ra treatment with that measured using ADC. The aim of the study was to investigate whether [^18^F]NaF PET/CT measurements of SUVmax or Ki have a potential role in assessing the response to ^223^Ra treatment in men with prostatic bone metastases.

## Methods

### The REASURE study

Thirty-nine men with a median age of 74.5 (IQR 72.1–79.5) from 3 oncology clinics in the United Kingdom were recruited between 27.05.2015 and 15.06.2017 and randomly assigned to receive either 55 or 88 kBq/kg of [^223^Ra]RaCl_2_ [11, 12]. Inclusion criteria were histologically confirmed adenocarcinoma of the prostate with castration-resistant disease, serum PSA of 2 ng/mL or more, ECOG performance status of 0 to 2, life expectancy > 6 months, and multiple skeletal metastases (≥ 2 hotspots) on bone scintigraphy within the previous 12 weeks. Exclusion criteria were visceral disease; lymphadenopathy greater than 1.5 cm in diameter; inadequate haematologic, kidney, or liver function; prior chemotherapy for castration-resistant prostate cancer; and any prior radionuclide therapy. After randomisation, up to six cycles of ^223^Ra therapy were administered intravenously at 4-week intervals. The trial was registered (ISRCTN17805587 [28]), approved by the National Health Service Health Research Authority Committee London–Surrey Borders Research Ethics Committee (14/LO/1385), and conducted in accordance with the principles of good clinical practice. All the participants gave written informed consent. Additional ethics approval was obtained for the present retrospective analysis of REASURE scan data.

### Imaging techniques

Response to treatment was monitored using measurements of ADC. Whole-body MRI scans were acquired on 1.5-T Siemens MAGNETOM Aera and MAGNETOM Avanto systems (Siemens Healthineers, Erlangen, Germany). Images were acquired from the skull base to mid-thigh, comprising diffusion-weighted and T1-weighted volume-interpolated breath-hold examination (VIBE) Dixon sequences, with matching field of view and slice thickness. Imaging was performed at baseline and at 4, 12, and 24 weeks after the first treatment. Up to five metastatic bone lesions were selected for monitoring in each participant. Full descriptions of the MRI scanning protocol were published in two earlier reports [12, 13].

Whole-body [^18^F]NaF PET/CT imaging was performed on the same day as the MRI scans. PET/CT studies were acquired on Siemens Biograph systems (Siemens Healthineers, Erlangen, Germany). Whole-body scans of 20 min duration with 7 bed positions were acquired from the vertex to mid-thighs starting 60 min after injection of 250 MBq of [^18^F]NaF tracer. A low-dose CT was performed for attenuation correction and image fusion. PET data were reconstructed using an ordered subset expectation maximisation algorithm. The same five target lesions identified on MRI were delineated as volumes of interest (VOI) on the baseline and follow-up PET/CT images using HERMES Gold software (Hermes Medical Solutions, Inc.) and values of SUVmax measured.

**Fig. 1:**
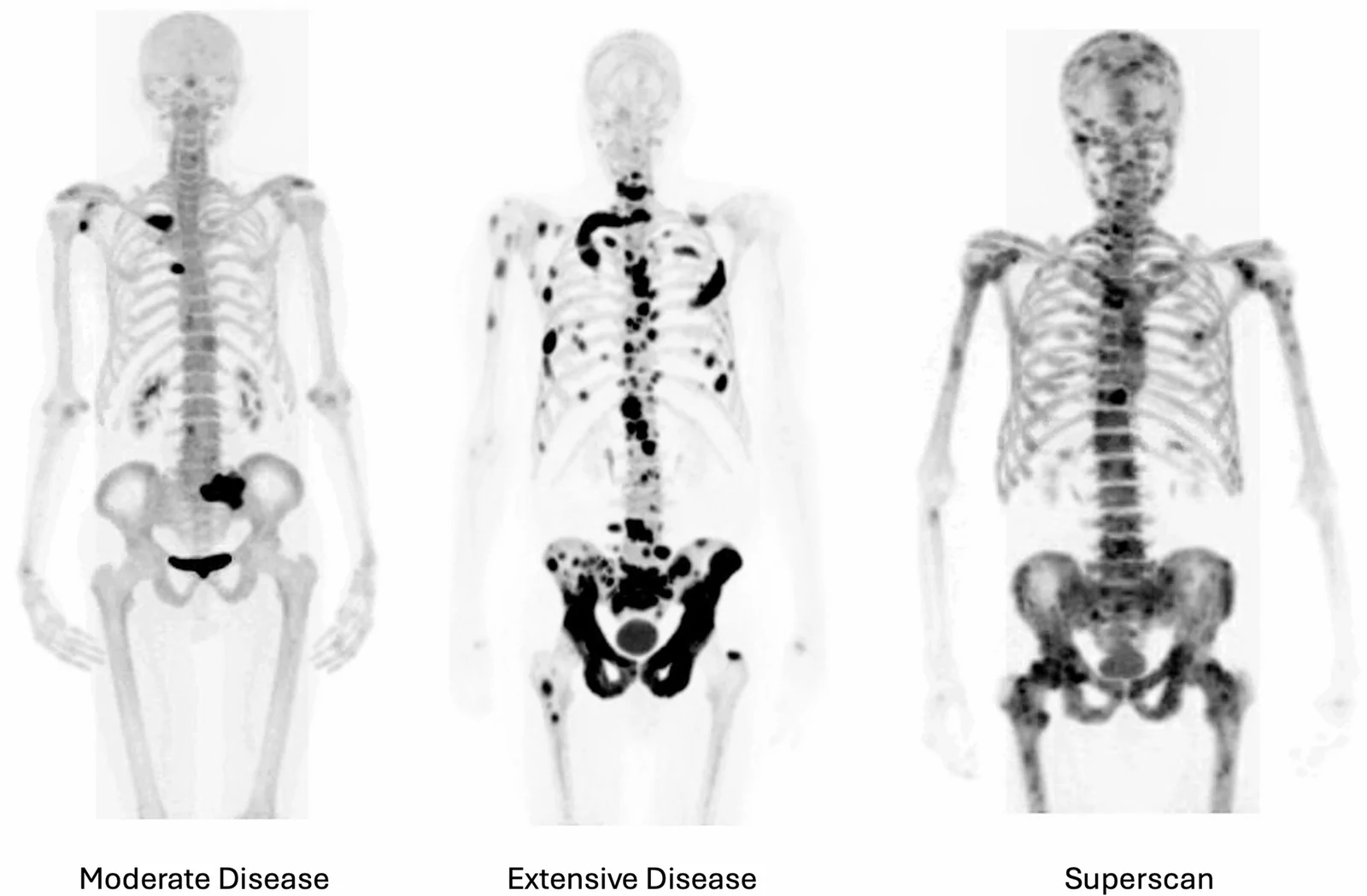
Examples of [^18^F]NaF PET/CT scans of men from the REASURE study with prostate cancer bone metastases categorised with either moderate disease, extensive disease, or a superscan

Conventionally, Ki is measured using the 60-minute dynamic scan method first described by Hawkins et al., with Ki evaluated using either compartmental analysis or from the slope of the Patlak plot [23]. However, this was not practical for the present retrospective study. Instead, Ki values were calculated from the SUVmax measurements of bone lesions on the whole-body [^18^F]NaF PET/CT scans **(**Fig. 1) using a novel surrogate method involving a measurement of whole-blood SUV in the left ventricle (LV SUV) **(Supplement Figure ****S1****)**. This was developed from the static scan method described by Siddique et al., which is based on measuring the final point of the Patlak plot and combining it with a population-based estimate of the intercept [29]. Ki is then derived from the slope of these two points. In Siddique’s method, information on the arterial input function is provided by a semi-population method, including the use of two or three venous blood samples to define the terminal exponential [30]. For the purposes of the present study, we further simplified the method to generate the Patlak plot using a single SUV measurement in the left ventricle acquired from the PET scan image. The **Supplement** available online gives a full description of how the LV SUV measurements were used to estimate the arterial input function and calculate Ki.

Each participant’s baseline [^18^F]NaF PET scan was assessed visually and graded as showing either a moderate degree of metastatic bone disease, extensive disease, or a superscan **(**Fig. 1**)**. Men in whom the extent of metastatic disease was judged too limited to significantly change the whole-body kinetics of the bone tracer were classified as having moderate disease [31]. Men in whom the extent of the lesions was judged likely to affect the tracer kinetics were classified as having extensive disease, while men with a superscan [32] were judged extremely likely to have depleted concentrations of circulating tracer likely to affect the relationship between Ki and SUVmax [31].

### Statistical analysis

All data were assessed for normality using the Shapiro-Wilk test, and parametric or non-parametric tests were used as appropriate. Measurements of LV SUV at baseline in men with moderate disease, extensive disease, and superscans were compared using the Kruskal-Wallis test followed by a post-hoc Dunn’s test. Data for baseline bone SUVmax, Ki, and the percentage changes in SUVmax between the baseline and the 12-week visits in the three PET scan categories were similarly compared, except that in these cases the data were analysed for the individual bone lesions monitored in the study.

The median percentage changes from baseline in LV SUV, ADC, SUVmax, and Ki at the 4-, 12-, and 24-week visits were plotted against the number of weeks since treatment began along with their 25th and 75th centiles. For ADC, SUVmax, and Ki, the data were plotted for the individual bone lesions monitored in the study. The statistical significance of the changes was assessed using the sign test, i.e., by comparing the number of positive and negative changes using binomial statistics.

Multilinear regression analysis (MLR) was used to investigate and compare the factors driving the changes in ADC and SUVmax. The analyses were performed for the baseline to week 4, baseline to week 12, and baseline to week 24 changes and for each time point compared the ADC and SUVmax changes in all the matched individual target lesions for which data were available. The first analysis compared the ability of the changes in SUVmax and Ki to predict the changes in ADC. We also compared the ability of the 4-week changes in SUVmax and ADC to predict the 12-week changes in ADC. Radium dose and baseline SUVmax were included as additional independent variables in the analyses. Similar MLR analyses were used to compare the ability of the changes in MRI ADC and Ki to predict the changes in SUVmax. For the MLR analyses, radium dose was expressed as either 0 or 1 for the 55 kBq/kg and 88 kBq/kg doses, respectively. Baseline SUVmax was expressed as the natural logarithm of the SUVmax measurement on the baseline scan. Changes in ADC, SUVmax, and Ki were expressed as the natural logarithm of the ratio of the follow-up/baseline measurements. The Statistics Kingdom and VassarStats websites were used for statistical analysis [33, 34]. A P-value of less than 0.05 was considered to indicate a statistically significant difference.

## Results

**Fig. 2:**
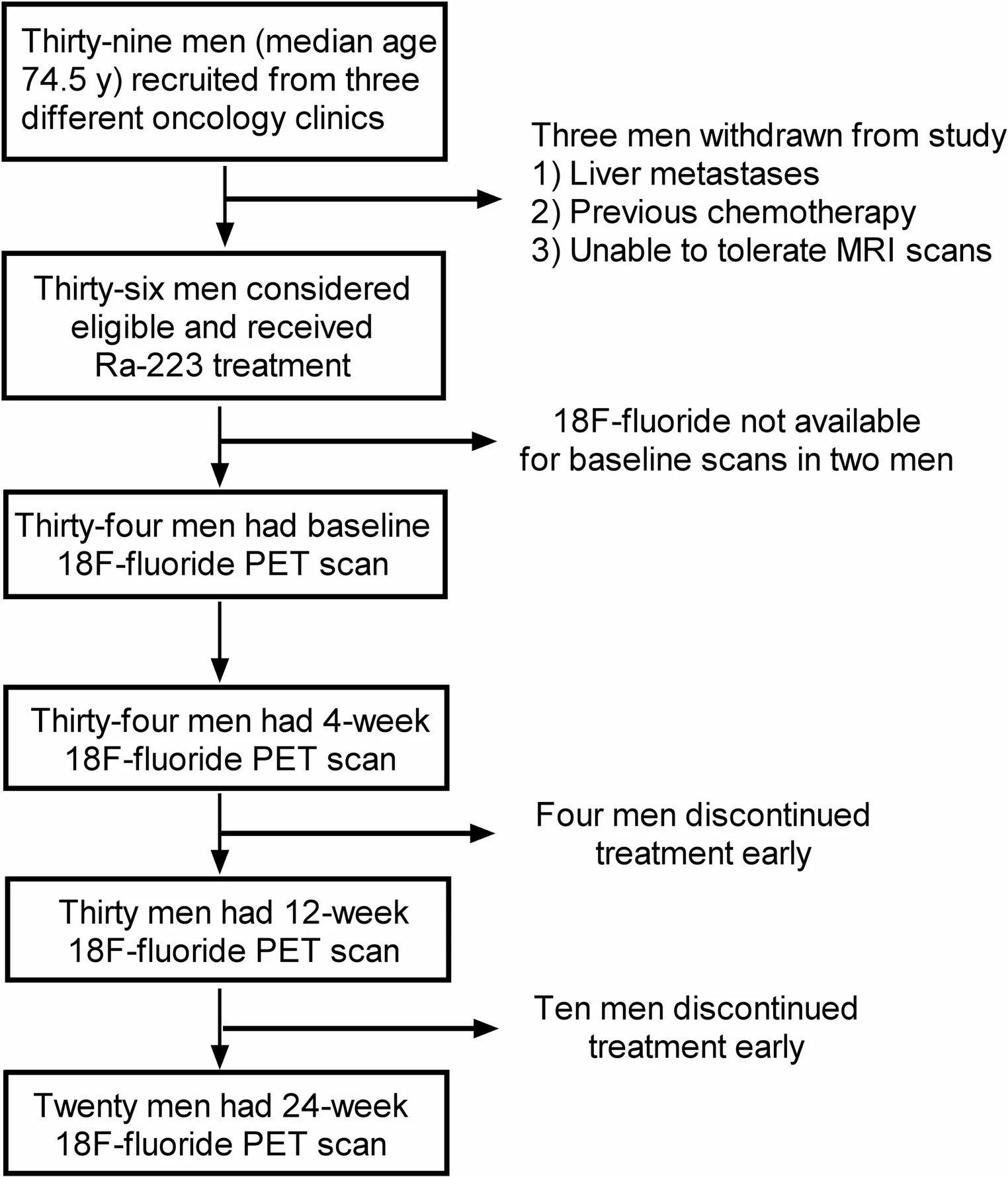
Flowchart explaining the number of participants at each stage of the study

Full demographics and tumour characteristics of the participants in the REASURE study have been published previously [12]. For the present analysis, ADC, SUVmax, and Ki data were available for 34 men. Of the 39 men originally recruited, 3 were withdrawn for clinical reasons previously explained [12, 13]. Two of the remaining 36 men were lost to the present study because the PET tracer was unavailable for their baseline visit. All 34 remaining men had baseline and 4-week follow-up scans; 30 had 12-week scans, and 20 had 24-week scans **(**Fig. 2**)**. The numbers of individual bone lesions measured at the three follow-up visits were 126, 110, and 69, respectively. Nineteen men received the 55 MBq/kg and fifteen the 88 MBq/kg treatment. One of the 3 men withdrawn from the study had baseline PET imaging but was later excluded after he was diagnosed with liver metastases. He is included in some of the present analysis because his PET/CT scan was a striking example of the effect of a superscan on the whole-body kinetics of bone tracer.

**Fig. 3:**
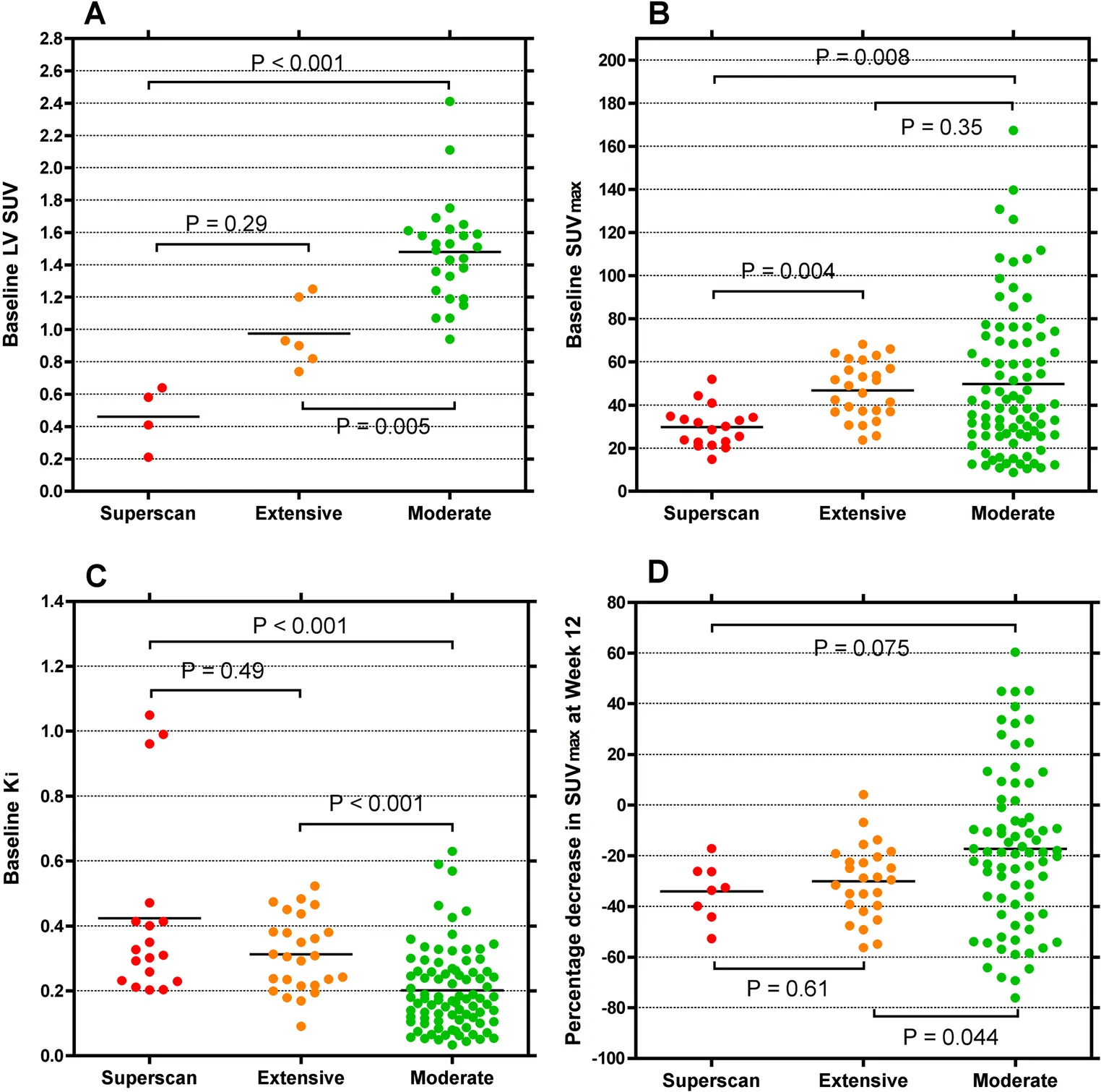
The effect of the extent of metastatic bone disease on the baseline measurements of **(A)** the standardised uptake value in the left ventricle (LV SUV) measured on the PET/CT scan image. **(B)** A similar plot of the SUVmax values measured in up to 5 individual bone metastases monitored in each participant. **(C)** Corresponding plot of the values of the bone metabolic flux (Ki) calculated from the bone lesion SUVmax and the LV SUV measurements. **(D)** Similar plot of the percentage change from baseline in the bone SUVmax measurements 12 weeks after the start of ^223^Ra treatment. P-values were calculated using the non-parametric Kruskal-Wallis test with Dunn’s correction for multiple comparisons

**Fig. 4:**
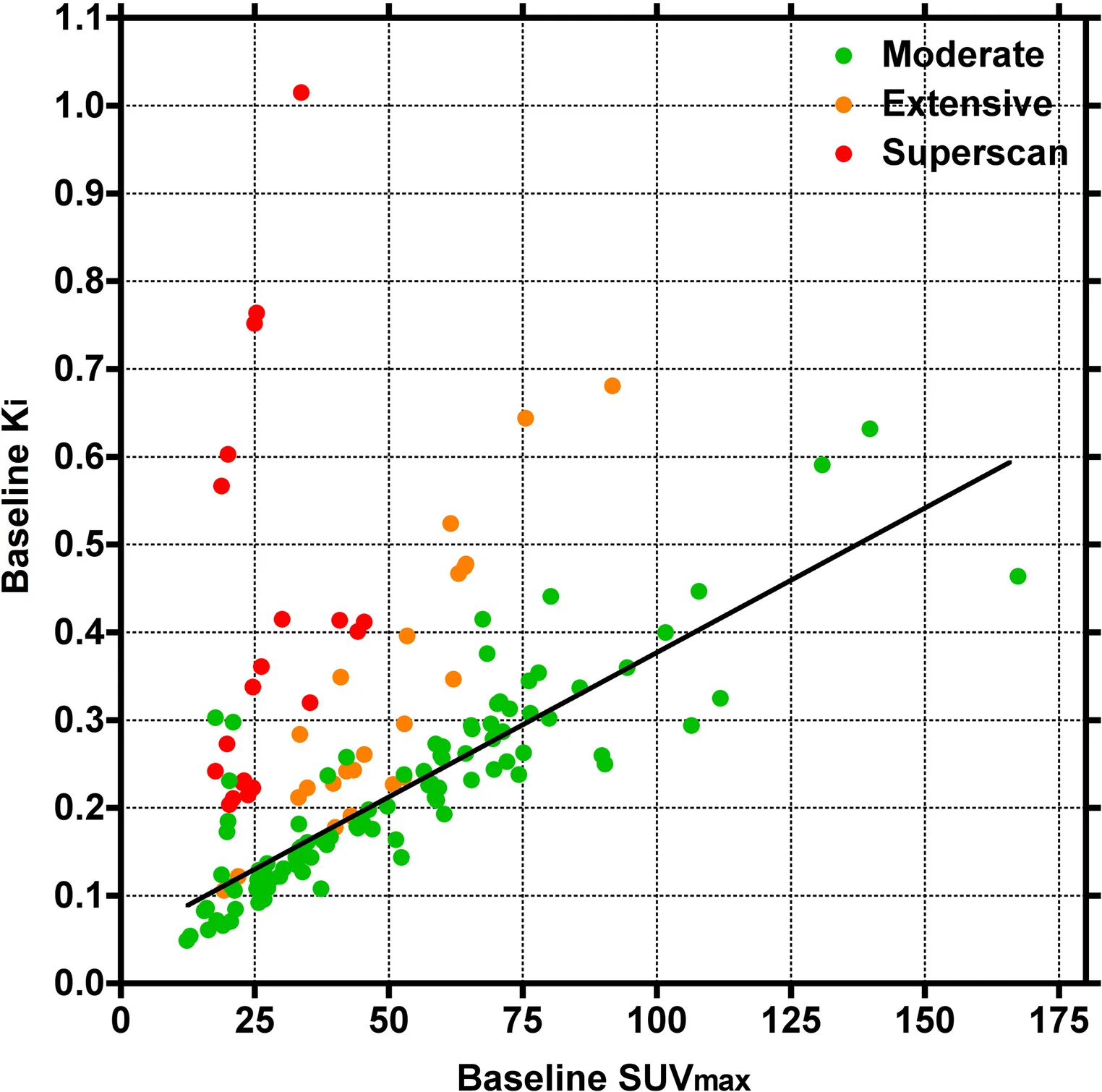
Plot of baseline Ki against baseline SUVmax for men with moderate disease, extensive disease, and superscans as defined in Fig. 1. The solid line shows the regression line fitted to the men with moderate disease (*r* = 0.93, *P* < 0.001)

The extent of metastatic bone disease had a significant influence on the baseline LV SUV, bone lesion SUVmax, and Ki measurements **(**Fig. 3**)**. Twenty-five men with a total of 87 monitored bone lesions included in the study were categorised as having moderate disease on their PET scans, 6 men with 26 monitored lesions as having extensive disease, and 4 men with 18 monitored VOIs were classed as superscans. Comparisons of LV SUV, SUVmax, Ki, and baseline to 12-week changes in SUV max between the three PET scan categories shown in Fig. 3 were assessed using the non-parametric Kruskal-Wallis test and gave P-values of < 0.001, 0.010, < 0.001 and 0.044 respectively **(**Figs. 3A-D**)**. Figure 3 shows the P-values for the comparisons between the different PET scan categories when Dunn’s test was applied. Men with superscans and extensive disease had significantly lower LV SUV compared with those with moderate disease **(**Fig. 3A**)**, and those with superscans had significantly lower bone SUVmax measurements than those with extensive or moderate disease (Fig. 3B**)**. When the Ki measurements were compared, these trends were reversed **(**Fig. 3C**)**. The extent of bone disease at baseline also influenced how many follow-up scans were performed before men were withdrawn from the study **(**Table 1**)**. The 30 men with 12-week scans included all 6 men with extensive disease but only 2 with superscans. The 20 men returning for 24-week scans included only 1 with extensive disease and no superscans. The extent of disease did not influence the percentage changes in bone SUVmax at the 12-week follow-up **(**Fig. 3D**)**. A plot of baseline Ki against baseline SUVmax for men with moderate disease, extensive disease, or superscans illustrates the significant effect that the extent of bone lesions across the skeleton has on the relationship between the two variables **(**Fig. 4**)**. The solid line shows the regression line fitted to the men with moderate disease (*r* = 0.93, *P* < 0.001).

**Table 1:** Number of men with moderate disease, extensive disease, and superscans at baseline and remaining in the study at weeks 4, 12, and 24

	Moderate Disease	Extensive Disease	Super Scans
Baseline	25	6	4†
Week 4	25	6	3
Week 12	22	6	2
Week 24	19	1	0

† Includes one man subsequently diagnosed with liver metastases and excluded from the study

**Fig. 5:**
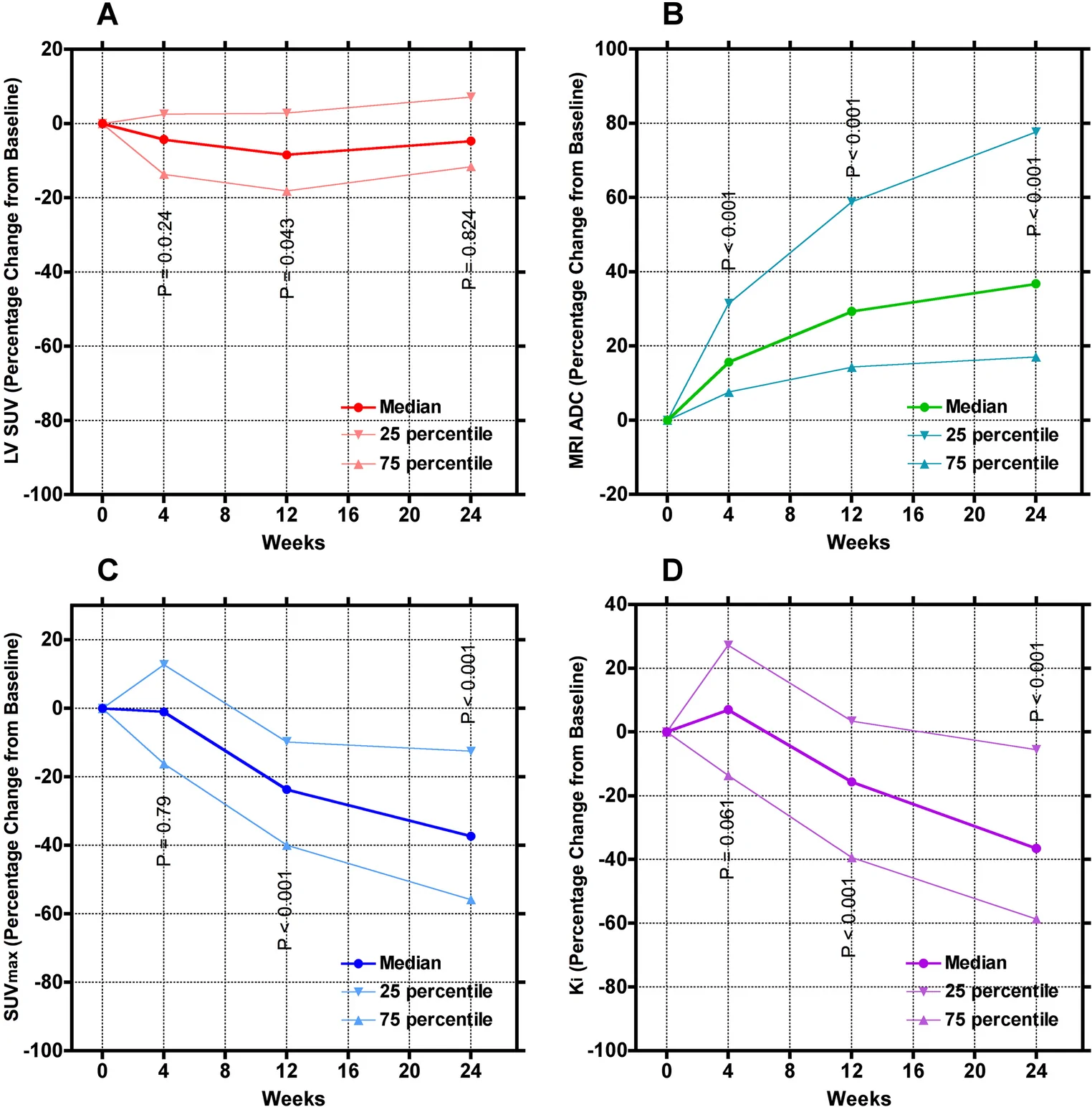
Percentage changes from baseline at 4, 12, and 24 weeks after the start of ^223^Ra treatment. Changes are shown as the median and interquartile range for the individual bone metastases in the men remaining in the study. **(A)** Results for the LV SUV measurements on the PET/CT scan images. **(B)** Measurements of the apparent diffusion coefficient (ADC) in individual bone metastases on the whole-body diffusion-weighted MRI scans. **(C)** Results for the SUVmax measurements in the same bone metastases measured on the [^18^F]NaF PET/CT scans. **(D)** Results for the measurements of bone metabolic flux (Ki) derived from the LV and bone SUVmax data. P-values were calculated using the sign test (i.e., by applying binomial statistics to the numbers of positive and negative changes, assuming equal numbers as the null hypothesis)

Figure 5 shows the percentage changes from baseline for LV SUV, ADC, SUVmax, and Ki measured on the 4-, 12-, and 24-week scans. Results are plotted as the median and interquartile range. At week 4, LV SUV decreased in 24 out of the 34 men (*P* = 0.024) and in 21 out of 30 men at week 12 (*P* = 0.043) **(**Fig. 5A**)**. For the ADC, SUVmax, and Ki measurements, the figures show the data for the individual bone lesions monitored during the study. Results for ADC showed highly significant increases at all follow-up times **(**Fig. 5B**)**, although the figure masks a trend in some bone lesions for the ADC measurement to decrease again by 24 weeks [11]. At week 4, ADC increased in 119 out of the 129 lesions for which ADC data was available; at week 12, in 112 out of 115 lesions; and at week 24, in 78 out of 84 lesions. When assessed as the mean change per patient, only 2 men out of 34 had a net decrease in ADC at week 4, and the 12- and 24-week changes were all positive. Therefore, there was a strong trend for the follow-up scans to show a positive response to ^223^Ra treatment as measured by ADC. In contrast to the ADC measurements, the changes in SUVmax at week 4 were not significant, with decreases in 65 out of 126 lesions (*P* = 0.79) **(**Fig. 5C**)**. However, 15 lesions in 5 men showed evidence of a bone flare phenomenon with increases in SUVmax of 30% or more at week 4 with a marked decrease by the 12- or 24-week time points. At week 12, 86 out of 110 lesions showed a negative response, and 57 out of 69 at week 24 (both *P* < 0.001). For Ki at week 4, 74 out of 126 lesions showed a positive change (*P* = 0.061) **(**Fig. 5D**)**. At weeks 12 and 24, the changes in Ki were like those for SUVmax.

**Fig. 6:**
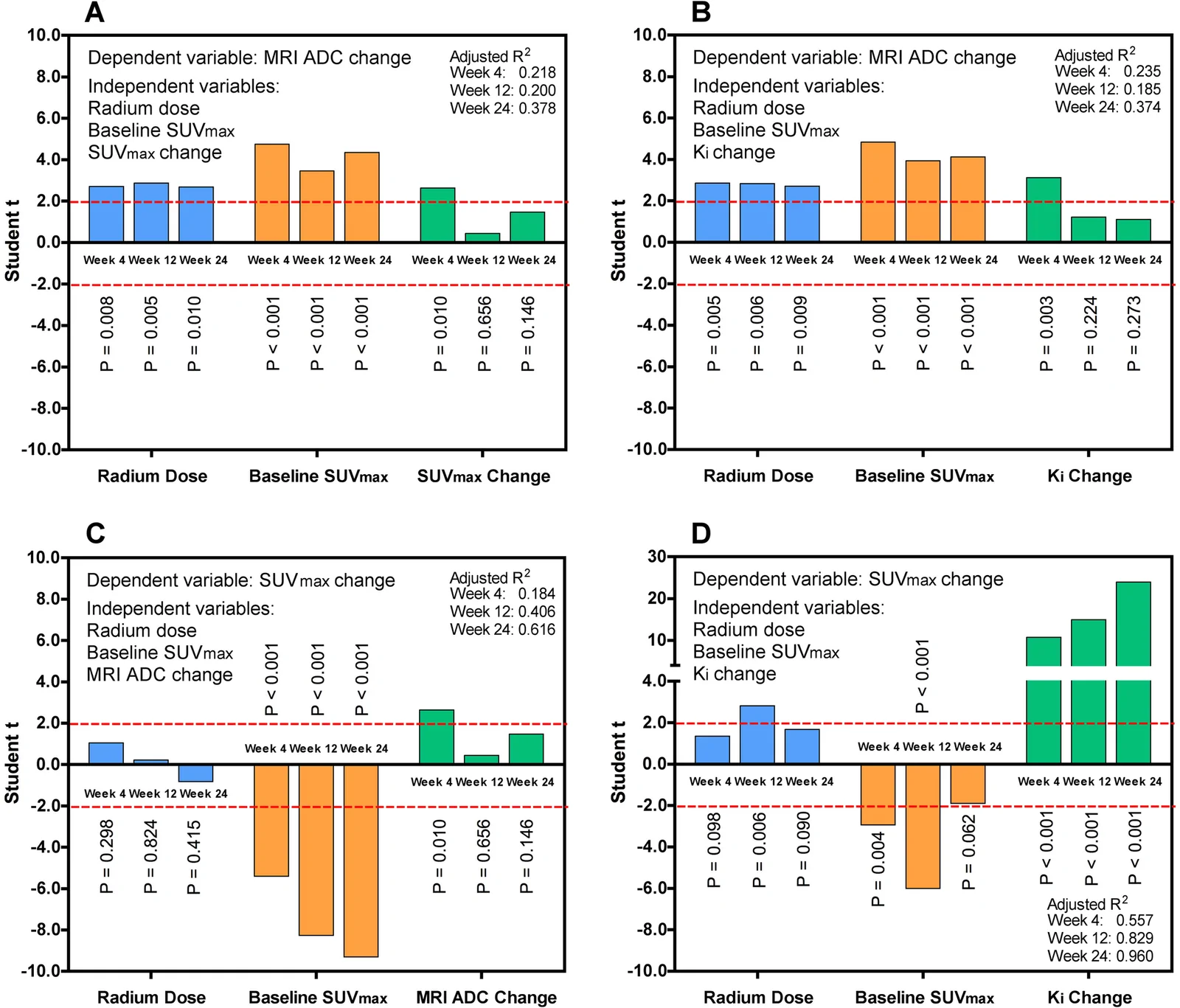
Plots of the Student-t values for the coefficients of the multilinear regression analyses summarised in Table 2. **(A)** Student t-values for the derivation of the changes in ADC from baseline using ^223^Ra dose (55 or 88 kBq/kg), baseline SUVmax, and the changes in SUVmax as independent variables. **(B)** Student t-values for the derivation of the changes in ADC from baseline using ^223^Ra dose, baseline SUVmax, and the changes in bone metabolic flux (Ki) as independent variables. **(C)** Student t-values for the derivation of the changes in SUVmax from baseline using ^223^Ra dose, baseline SUVmax, and the changes in ADC as independent variables. **(D)** Student t-values for the derivation of the changes in SUVmax from baseline using ^223^Ra dose, baseline SUVmax, and the changes in Ki as independent variables. The red dashed lines show the 95% confidence limits for a statistically significant result. ^223^Ra dose is expressed as either 0 or 1 for the 55 kBq/kg and 88 kBq/kg doses, respectively. Baseline SUVmax is expressed as the natural logarithm of the SUVmax measurement on the baseline scan. Changes in ADC, SUVmax, and Ki are expressed as the natural logarithms of the ratio of the follow-up/baseline measurements

The results of the multilinear regression analyses are shown in Table 2 and Fig. 6. Table 2A and Table 2B compare, respectively, the regression coefficients, standard errors, and P-values at the 4-, 12-, and 24-week visits for the changes in SUVmax **(**Table 2A**)** and Ki **(**Table 2B**)** to predict the changes in ADC. As noted above, ^223^Ra dose and baseline SUVmax were included as independent variables in the MLR analyses alongside the changes in SUVmax and Ki. Figure 6A and B illustrate the same analyses visually by plotting the statistical significance of the regression coefficients in terms of their Student’s t-values. The MLR coefficients in the two tables and the Student’s t-values in the two figures are similar for SUVmax and Ki. In both analyses, baseline SUVmax and ^223^Ra dose are the principal factors predicting the changes in ADC, while the changes in SUVmax and Ki are not statistically significant variables at the 12- or 24-week visits. The 4-week changes in SUVmax were no better at predicting the 12-week changes in ADC than they were the 4-week changes (Student’s t = 1.49, *P* = 0.14). However, the 4-week changes in ADC were a highly significant predictor of the 12-week changes in ADC (Student’s t = 9.66, *P* < 0.001). This result reflects the relatively high correlation between the two measurements (*r* = 0.75).

**Table 2:** ^223^Ra dose is expressed as either 0 or 1 for the 55 kBq/kg and 88 kBq/kg doses, respectively. Baseline SUVmax is expressed as the natural logarithm of the SUVmax measurement on the baseline scan. Changes in ADC, SUVmax, and Ki are expressed as the natural logarithms of the ratio of the follow-up/baseline measurements. **A** Results of multiple linear regression analysis to predict the change in ADC using ^223^Ra dose, baseline SUVmax, and the change in SUVmax as independent variables. **B** Results of multiple linear regression analysis to predict the change in ADC using ^223^Ra dose, baseline SUVmax, and the change in bone metabolic flux (Ki) as independent variables. **C** Results of multiple linear regression analysis to predict the change in SUVmax using ^223^Ra dose, baseline SUVmax, and the change in ADC as independent variables. **D** Results of multiple linear regression analysis to predict the change in SUVmax using ^223^Ra dose, baseline SUVmax, and the change in bone metabolic flux (Ki) as independent variables

A		Week 4	Week 12	Week 24
^223^Ra dose	Coefficient	0.0634	0.1047	0.1419
	Standard error	0.0234	0.0366	0.0530
	P-value	P = 0.008	P = 0.005	P = 0.010
Baseline SUVmax	Coefficient	0.0951	0.1266	0.2464
	Standard error	0.0200	0.0366	0.0566
	P-value	P < 0.001	P < 0.001	P < 0.001
SUVmax change	Coefficient	0.1471	0.0285	0.1118
	Standard error	0.0557	0.0638	0.0760
	P-value	P = 0.010	P = 0.656	P = 0.146
B		Week 4	Week 12	Week 24
^223^Ra dose	Coefficient	0.0657	0.1082	0.1453
	Standard error	0.0230	0.0382	0.0537
	P-value	P = 0.005	P = 0.006	P = 0.009
Baseline SUVmax	Coefficient	0.0927	0.1340	0.2285
	Standard error	0.0191	0.0340	0.0555
	P-value	P < 0.001	P < 0.001	P < 0.001
Ki change	Coefficient	0.1400	0.0687	0.0799
	Standard error	0.0448	0.0562	0.0722
	P-value	P = 0.003	P = 0.224	P = 0.273
C		Week 4	Week 12	Week 24
^223^Ra dose	Coefficient	0.0396	0.0128	-0.0732
	Standard error	0.0379	0.0577	0.0891
	P-value	P = 0.298	P = 0.824	P = 0.415
Baseline SUVmax	Coefficient	-0.1667	-0.3781	-0.6296
	Standard error	0.0309	0.0458	0.0677
	P-value	P < 0.001	P < 0.001	P < 0.001
MRI ADC change	Coefficient	0.3670	0.0659	0.2883
	Standard error	0.1390	0.1475	0.1959
	P-value	P = 0.010	P = 0.656	P = 0.146
D		Week 4	Week 12	Week 24
^223^Ra dose	Coefficient	0.0364	0.0877	0.0464
	Standard error	0.0270	0.0311	0.0277
	P-value	P = 0.181	P = 0.006	P = 0.098
Baseline SUVmax	Coefficient	-0.0661	-1.669	-0.0544
	Standard error	0.0225	0.0278	0.0287
	P-value	P = 0.004	P < 0.001	P = 0.062
Ki change	Coefficient	0.5668	0.6865	0.8947
	Standard error	0.0527	0.0458	0.0373
	P-value	P < 0.001	P < 0.001	P < 0.001

 Tables 2C and 2D and Fig. 6C and D show the results of similar MLR analyses that compare, respectively, the ability of changes in ADC and Ki to predict the changes in SUVmax. Baseline SUVmax is the principal factor predicting the change in SUVmax in Fig. 6C, and the changes in Ki are the principal factor in Fig. 6D.

## Discussion

In this study, we analysed MRI and PET/CT imaging data from the REASURE study and used multilinear regression analysis to compare the ability of the changes in SUVmax and Ki measured by [^18^F]NaF PET/CT to predict the changes in ADC in men receiving ^223^Ra palliation for prostatic bone metastases and also to investigate how the changes in ADC and SUVmax were influenced by other confounding factors such as the administered activity of ^223^Ra and the baseline SUVmax of the metastatic lesions.

Previous studies have shown that powerful and extensive changes in bone metabolism due to metabolic or metastatic bone disease or their treatment can have a profound effect on the pharmacokinetics of bone imaging radiopharmaceuticals [26, 31, 35]. The present study confirmed that in men with extensive metastatic bone disease from prostate cancer, the burden of bone involvement had an important influence on the kinetics of the [^18^F]NaF tracer. Multiple widespread areas of osteosclerotic metastatic disease that involve much of the skeleton act as a powerful sink that captures much of the available tracer before it can be laid down in normal bone or excreted through the kidneys. In contrast, in men with a limited degree of metastatic disease, the handling of bone tracer differs little from that in normal subjects [31].

Measurements of Ki correct SUVmax measurements for the relative availability of bone tracer in the circulation and provide an index that more reliably reflects the degree of osteoblastic activity at the measurement site [26]. In the present study, the trend of the Ki measurements with the extent of metastatic disease was the reverse of that shown by the SUVmax measurements. In consequence, for the men with superscans and extensive disease, there was a decoupling between the two measurements and a significant departure from the linear relationship found in those with moderate disease, as illustrated in Fig. 4. It follows that in men with moderate disease, Ki is unlikely to outperform SUVmax as a marker of response to treatment given the additional measurements and assumptions required in its estimation. In the REASURE study, men with moderate disease accounted for 70% of participants at baseline and 95% of those finishing the study (Table 1). The results of the present study therefore principally reflect the trends in these men.

The measurements of the percentage change in ADC showed a progressive increase throughout the study. ADC increased in 92% of the individual lesions at the 4-week visit, in 97% at 12 weeks, and in 93% at 24 weeks. At the 4-week visit, only 2 participants out of 34 showed a decrease in the average percentage change in ADC across all sites monitored in the study, and none at the 12- or 24-week visits. It is therefore arguable that almost all participants showed evidence of some degree of positive response to radium-223 treatment as monitored by the ADC measurements.

In contrast to the ADC measurements, there was no significant change in SUVmax at 4 weeks after the start of treatment. However, by 12 weeks and 24 weeks there were median decreases of -24% and -37%, respectively. The lack of response in SUVmax at 4 weeks is a striking difference from the ADC data. It is likely that a bone flare in some men is masking what would otherwise be a net decrease in SUVmax at the 4-week time point [19–22]. It is also likely that the process of ongoing secondary mineralisation in new bone laid down in the weeks immediately preceding the first cycle of radium treatment contributes to the lack of an early response. Despite the lack of a systematic response at the 4-week visit, at the 12- and 24-week visits, 83% and 85% of men, respectively, showed a positive response, as reflected by a decrease in their average SUVmax measurements from baseline. There is less data available to assess the changes in LV SUV because only a single measurement is available at each follow-up visit. Small decreases were found at the 4- and 12-week visits, but these were only marginally significant. For Ki, the changes were similar to those for SUVmax.

Multilinear regression analysis was performed to investigate the importance of ^223^Ra dose, baseline SUVmax, and the changes in either SUVmax or Ki on the post-therapy PET scans as factors explaining the changes in ADC. Also, the importance of ^223^Ra dose, baseline SUVmax, and the changes in either ADC or Ki for explaining the post-therapy changes in SUVmax. A striking finding of this analysis was the strong dependence of both the ADC and SUVmax responses to treatment on the baseline SUVmax. The former relationship was previously reported by Donners et al. using a different analysis [13].

These findings suggest a correlation between ^223^Ra uptake in bone lesions and the SUVmax measurement on the [^18^F]NaF PET/CT scan. This is predictable given that radium is an alkaline earth metal whose metabolism in the body is similar to that of calcium, strontium, and bone imaging agents such as [^99m^Tc]MDP [36, 37]. ^223^Ra uptake in bone lesions is therefore expected to reflect the uptake seen on the [^18^F]NaF PET/CT scan. A bone metastasis with a higher SUVmax will have a greater uptake of ^223^Ra, leading to a correspondingly greater radiation dose to tumour cells as well as to local osteoclasts and osteoblasts, and therefore having a greater effect on the follow-up ADC and SUVmax measurements.

The randomisation of REASURE study participants to either the 55 or 88 kBq/kg ^223^Ra dose had a smaller effect on the changes in ADC than that associated with baseline SUVmax. This is expected given that the ratio of the two treatment doses (88/55 = 1.6) is much smaller than the range of baseline SUVmax values (8.7 to 167.4). The direction of the change in ADC with ^223^Ra dose in Fig. 6A and B is consistent with the changes due to baseline SUVmax, i.e., a higher dose leads to a greater increase in ADC. However, the same consistency is not seen in Fig. 6C and D, where the coefficients for ^223^Ra dose to predict the changes in SUVmax are either not significant or trend in the opposite direction to the coefficients for baseline SUVmax.

Another feature of the results in Fig. 6A and B is that, at the 12- and 24-week visits, neither the change in SUVmax nor the change in Ki are significant predictors of the changes in ADC. Furthermore, the weak trends seen are in the opposite direction to those expected. The lack of any significant relationship indicates that the ADC and PET responses have different mechanisms, with the ADC changes reflecting the effect of the ^223^Ra alpha rays on tumour cells and changes in the SUVmax and Ki reflecting the effect on osteoclasts and osteoblasts.

A further feature of the results illustrated by Fig. 6D is that Ki change was by far the dominant factor for explaining the changes in SUVmax at all three follow-up visits. This finding is expected given the strong correlation between the Ki and SUVmax measurements seen in Fig. 4 for men with moderate disease and the dominance of this group of men among the participants completing the study. In men with moderate disease there is little scope for decoupling between the two measurements because the extent of the metastatic disease has minimal effect on the plasma clearance curve of the bone tracer. Hence, it is not surprising that the results in Fig. 6A and B appear so similar.

The findings of this study showed that neither the changes in SUVmax nor the changes in Ki measured by [^18^F]NaF PET/CT at the 12- and 24-week visits predicted the response to ^223^Ra treatment measured by the changes in ADC. In addition, the bone flare phenomenon and ongoing secondary mineralisation prevented the changes in the PET measurements at the 4-week visit from being useful in this regard. Figure 4 shows the strong correlation between the SUVmax and Ki measurements in men with moderate disease, and Fig. 6D shows how this correlation is reflected in the highly statistically significant coefficients in the MLR analysis. The failure to see a similar effect in Fig. 6A and B reinforces the evidence for an almost complete decoupling of the processes driving the changes in the MRI and PET/CT measurements. There is strong evidence that the changes in ADC are a direct measurement of the tumoricidal effect of alpha rays from ^223^Ra laid down in bone metastases on adjacent metastasised prostate cancer cells [38]. The decoupling seen in Fig. 6A and B suggests that the changes in SUVmax may be driven wholly or in part by a different mechanism to that causing the changes in ADC, i.e., that the changes in the follow-up PET scans are due to the action of ^223^Ra alpha rays on osteoblastic activity rather than any direct cytotoxic effect on tumour cells.

We acknowledge that this study has several limitations. These include the small size of the study, with only 20 participants completing their end-of-treatment imaging studies. A larger study might have demonstrated a statistically stronger relationship between the changes in ADC and SUVmax in response to treatment. In addition, only 10 men with extensive disease or superscans were enrolled in the study, and only one of these completed the study. These latter issues limited the value of the study for studying the effect of disease extent on the response to ^223^Ra treatment and the differences between SUVmax and Ki measurements as variables for monitoring response to treatment. We also acknowledge the limited accuracy of the estimates of Ki made using the LV SUV measurements. Ideally, a 60-minute dynamic scan over the heart from the moment of injection would have provided more reliable data on the plasma clearance curve of the [^18^F]NaF tracer, especially in the men with extensive disease in whom plasma clearance is more rapid than normal. Further studies are required to validate the use of a single LV SUV measurement to reproduce measurements of Ki made using the conventional 60-minute dynamic scan.

In conclusion, this study has demonstrated how the plasma clearance of bone tracers is accelerated in patients with extensive sclerotic metastases, thereby also reducing SUVmax measurements made using PET/CT. Since [^18^F]NaF and [^223^Ra]RaCl_2_ are laid down in bone by broadly similar mechanisms, it is reasonable to infer that the uptake of ^223^Ra will be affected in a similar manner. The study has shown an important difference in the responses of the ADC and SUVmax measurements 4 weeks after the start of ^223^Ra treatment compared with the 12- and 24-week time points. This, and the lack of a statistically significant relationship between the changes in SUVmax and ADC, suggests that these changes may be driven by different mechanisms. SUVmax measurements may largely reflect the changes in osteoblastic activity at the measurement site and do not appear to be strongly related to the changes in ADC associated with the tumoricidal effects of ^223^Ra treatment. While high values of SUVmax measured on a baseline [^18^F]NaF PET/CT scan are predictive of a good response to ^223^Ra treatment, any improvements seen on follow-up scans should not be interpreted as evidence of tumour regression.

## Supplementary Information

Below is the link to the electronic supplementary material.


Supplementary Material 1 (DOCX 835 KB)


## Data Availability

The datasets generated and analysed during the current study are available from the corresponding author on reasonable request.

## Declarations

### Guarantor

The scientific guarantor of this publication is Professor Glen Blake.
