# Supplementary material for: Does 18F-sodium fluoride PET/CT have a role in the assessment of response to 223Ra therapy in men with prostatic bone metastases?
Source: Eur J Nucl Med Mol Imaging. 2026 Jun 20;53(11):6536–48. doi: 10.1007/s00259-026-08009-8 (PMC13526093; doi:10.1007/s00259-026-08009-8)
Supplement: Supplementary file 1 [file 259_2026_8009_MOESM1_ESM.docx]

**Does ^18^F-sodium fluoride PET/CT have a role in the assessment of response to ^223^Ra therapy in men with prostatic bone metastases?**

Glen M. Blake^1^, Ricardo Donners^2^, Amelia E.B. Moore^3^, Holly Tovey^4^, Aude Espinasse^4^, Matthew D. Blackledge^4^, Sue Chua^5^, Yong Du^5^, Emma Hall^4^, Dow-Mu Koh^4,5^, Emilia Nuzzaci^4^, Nina Tunariu^4,5^, Christopher C. Parker^4,5^, Gary J.R. Cook^3^

1. Department of Biomedical Engineering, School of Biomedical Engineering & Imaging Sciences, King’s College London, St Thomas’ Hospital, London, UK.
2. Department of Radiology, University Hospital Basel, Petersgraben 4, 4031 Basel, Switzerland.
3. Department of Cancer Imaging, School of Biomedical Engineering & Imaging Sciences, King's College London, St Thomas' Hospital, London, UK.
4. The Institute of Cancer Research, 15 Cotswold Road, Sutton SM2 5NG, UK.
5. Royal Marsden Hospital, Downs Road, Sutton SM2 5PT, UK.

**SUPPLEMENTAL MATERIAL**

**Measurement of Bone Metabolic Flux (Ki) using PET/CT Imaging of the Left Ventricle**

**The Bone Sub-Study**

The Bone Sub-Study was an extension of the main REASURE study [1,2] with the aim of using the [^18^F]NaF PET scans to measure bone metabolic flux (Ki, also referred to as bone plasma clearance) [3,4] and compare and contrast the response to ^223^Ra treatment with the response measured using maximum standardised uptake values (SUVmax) and the MRI apparent diffusion coefficient (ADC) [5]. As originally planned, the Bone Sub-Study entailed taking blood samples immediately before and after the baseline and 12-week whole-body PET scans to measure the [^18^F]NaF plasma concentrations at approximately 60 and 90 minutes after injection. It was planned to estimate Ki values using the static scan method of Siddique et al. [4,6]. Men enrolled in the REASURE study were invited to participate in the Bone Sub-Study and to sign an additional consent form approved by the Ethics Committee. Twenty-five out of the 39 men in the REASURE study consented to join the Sub-Study. However, at the end of the study, data for only 17 men was available for statistical analysis. Four men withdrew from the study before their 12-week scans. Two men did not have baseline scans due to the [^18^F]NaF tracer being unavailable. In two other men, either the baseline or 12-week [^18^F]NaF plasma concentrations exceeded the upper limit of normal male values observed in a previous study [7] and were not consistent with those measured for the baseline or follow-up scan, and the results were considered unreliable.

To increase the number of men whose data was available for statistical analysis, a decision was made to base the Ki calculations on a measurement of [^18^F]NaF whole-blood concentration in the left ventricle (LV). Additional Ethics Committee approval was obtained for a retrospective reanalysis of the PET scans in the men who had consented to future analysis of their scans. This included all the remaining men in the REASURE study. This change in protocol doubled the number of men available for the baseline to 12-week analysis and expanded the scope of the study by allowing inclusion of the baseline to 4-week and baseline to 24-week data.

**PET Scan Measurements of [^18^F]NaF Concentration in the Left Ventricle**

Two raters experienced in PET/CT scan image analysis independently placed volumes of interest within the LV to measure the mean whole-blood SUV **(Figure S1)**. The average scan start time was 65 minutes after tracer injection (standard deviation 6 min, range 56 to 83 min). We assumed that the effective time for imaging the LV was halfway through the 20-minute PET scan, giving an average LV SUV measurement time of 75 minutes after injection. The average LV SUV measured by each rater was 0.94 and 0.97, respectively, and the individual results were well correlated (**Figure S2A**, r = 0.976). A Bland-Altman plot gave a bias of 0.028 and 95% limits of agreement (LOA) of -0.105 to +0.161 (**Figure S2B**). In view of the small bias relative to the LOA, the raters’ measurements were averaged for further analysis.

Figure S1. [^18^F]NaF PET/CT images showing the placement of the left ventricle (LV) volume of interest (VOI) for the measurement of LV SUV. (A) Transaxial CT image through the middle of the left ventricle showing the placement of the LV VOI. (B) Corresponding slice through the [^18^F]NaF PET image. (C) Fusion image of (A) and (B). (D) Corresponding whole-body [^18^F]NaF PET image. (E) Coronal CT image through the middle of the left ventricle showing the placement of the LV VOI. (F) Corresponding slice through the [^18^F]NaF PET image. (G) Fusion image of (E) and (F).

Figure S2. (A) Plot of left ventricle standardised uptake values (LV SUV) of Rater 2 vs. Rater 1.

(B) Bland-Altman plot of the data in (A).

**Correction of LV Whole-Blood SUV to LV Plasma SUV**

The convention in [^18^F]NaF PET/CT studies of bone metabolic flux is to measure plasma clearance rather than whole-blood clearance of tracer [3]. For the present study, we derived plasma concentrations by multiplying the LV whole-blood concentrations by a factor of 1.34. This latter figure was the mean ratio of plasma to whole-blood concentration obtained in a recently published [^18^F]NaF PET/CT study of the effect of a change of treatment regime on bone turnover at the hip and spine in 32 men aged 40-65 years living with HIV [7]. The average ratio (SD) found in 224 independent blood samples was 1.342 (0.062). There was no clinically significant variation in this ratio with the time after injection that the blood sample was taken [30 min: 1.349 (0.069); 60 min: 1.337 (0.056); 90 min: 1.340 (0.061)] or the number of weeks since the baseline PET scan [baseline: 1.334 (0.055); 24 weeks: 1.345 (0.066); 48 weeks: 1.350 (0.066)]. As a check, the ratios of the direct blood sample measurements of [^18^F]NaF plasma concentration measured for the REASURE Bone Sub-Study to the estimates obtained by multiplying the LV SUV measurements by a factor of 1.34 were plotted for the 44 scan visits for which both measurements were available **(Figure S3)**.

Figure S3. Ratios of the geometric mean of the 65- and 85-minute [^18^F]NaF plasma concentrations measured in the original Bone Sub-Study protocol to the 75-minute concentrations estimated by using the PET/CT scans to measure the whole blood concentration in the left ventricle. The latter were multiplied by a factor of 1.34 to convert whole-blood data into plasma concentrations. The three brown dots are far outliers believed to be due to erroneous plasma concentration measurements.

The direct blood sample measurements plotted here are the geometric mean of the data obtained immediately before and after the 20-minute PET scans on the assumption that the plasma concentration is falling exponentially. Three results shown in brown are far outliers, including the men mentioned above who were excluded from the original sub-study for this reason. The green dots have ratios that are generally slightly greater than 1.0. This may reflect the fact that, in practice, the [^18^F]NaF plasma clearance curves are multi-exponential, while taking the geometric mean of the 65- and 85-minute blood samples assumes a single exponential fit in this time range.

**Calculation of Bone Metabolic Flux (Ki)**

Estimates of Ki were made using the static scan method of Siddique et al. based on the following equation [6]:

Measurements of the tissue and plasma concentrations of [^18^F]NaF tracer (*C_tissue_* and *C_plasma_*) were expressed in units of SUV. *C_plasma_* (*T*) is the measurement of the left ventricle SUV assumed to be measured 10 minutes after the start time of the PET scan. The SUV measurements in the bone lesions are all assumed to refer to the same time point. *V*_0_ is the volume of distribution of the [^18^F]NaF tracer in the bone lesion, and for this study the value of 0.43 published by Siddique et al. was adopted [6]. The integral of the plasma concentration from the time of injection to the midpoint of the PET/CT scan was estimated in two stages. First, the LV SUV measurement at time T was extrapolated back to 60 minutes after injection, assuming an exponential plasma clearance curve with a 1/e time of 120 minutes. This was the average time found in the study of men with HIV referred to above [7]. Secondly, the area under the plasma clearance curve from 0 to 60 min was estimated by multiplying the 60-minute plasma concentration by a factor of 128.8. This was the average ratio found from an analysis of the direct arterial sampling study published by Cook et al. [8]. Individual values in the nine subjects in that study varied from 120.5 to 133.3.

**References**

1. Parker C, Tunariu N, Tovey H, Alonzi R, Blackledge MD, Cook GJR, et al. Radium-223 in metastatic castration-resistant prostate cancer: whole-body diffusion-weighted magnetic resonance imaging scanning to assess response. JNCI Cancer Spectr. 2023 Oct 31;7(6):pkad077.
2. Donners R, Tunariu N, Tovey H, Hall E, Chua S, Cook G, et al. The value of baseline 18F‑sodium fluoride and 18F‑choline PET activity for identifying responders to radium‑223 treatment in castration‑resistant prostate cancer bone metastases. Eur Radiol. 2024 Feb;34(2):1146-1154.
3. Hawkins RA, Choi Y, Huang S-C, Hoh CK, Dahlbom M, Schiepers C, et al. Evaluation of the skeletal kinetics of fluorine-18-fluoride ion with PET. J Nucl Med 1992 May;33(5):633-642.
4. Puri T, Frost ML, Moore AEB, Cook GJR, Blake GM. Input function and modeling for determining bone metabolic flux using [^18^F] sodium fluoride PET imaging: A step-by-step guide. Med Phys. 2023 Apr;50(4):2071-2088.
5. Scialpi M, Martorana E, Scialpi P, D'Andrea A, Mancioli FM, Mignogna M, et al. MRI apparent diffusion coefficient (ADC): A biomarker for prostate cancer after radiation therapy. Turk J Urol. 2021 Nov;47(6):448-451.
6. Siddique M, Blake GM, Frost ML, Moore AE, Puri T, Marsden PK, Fogelman I. Estimation of regional bone metabolism from whole-body18F-fluoride PET static images. Eur J Nucl Med Mol Imaging. 2012 Feb;39(2):337-43.
7. Moore AEB, Burns JE, Sally D, Milinkovic A, Krokos G, John J, et al. Bone turnover change after randomized switch from tenofovir disoproxil to tenofovir alafenamide fumarate in men with HIV. AIDS. 2024 Mar 15;38(4):521-529.
8. Cook GJ, Lodge MA, Marsden PK, Dynes A, Fogelman I. Non-invasive assessment of skeletal kinetics using fluorine-18 fluoride positron emission tomography: evaluation of image and population-derived arterial input functions. Eur J Nucl Med. 1999 Nov;26(11):1424-1429.
